# Infliximab for the Treatment of Inflammatory Labyrinthitis: A Retrospective Cohort Study

**DOI:** 10.3390/jcm12134350

**Published:** 2023-06-28

**Authors:** Cassandre Djian, Karine Champion, Nicolas Lai, Ludovic Drouet, Blanca Amador Borrero, Audrey Depond, Stéphane Mouly, Clément Jourdaine, Philippe Herman, Michael Eliezer, Charlotte Hautefort, Damien Sène

**Affiliations:** 1 Department of Otolaryngology, Hôpital Lariboisière, Assistance Publique—Hôpitaux de Paris, 75010 Paris, France; nicolas.lai@aphp.fr (N.L.); clement.jourdaine@aphp.fr (C.J.); philippe.herman@aphp.fr (P.H.); charlotte.hautefort@aphp.fr (C.H.); 2 Department of Internal Medicine, Hôpital Lariboisière, Assistance Publique—Hôpitaux de Paris, 75010 Paris, France; karine.champion@aphp.fr (K.C.); blanca.amador-borrero@aphp.fr (B.A.B.); audrey.depond@aphp.fr (A.D.); stephane.mouly@aphp.fr (S.M.); damien.sene@aphp.fr (D.S.); 3 Department of Internal Medicine, Saint Joseph Hospital, 75014 Paris, France; ludovic.drouet@aphp.fr; 4 Faculté de Médecine, Paris Cité University, 75006 Paris, France; 5 Department of Neuroradiology, Hôpital Lariboisière, Assistance Publique—Hôpitaux de Paris, 75010 Paris, France; michael.eliezer@aphp.fr

**Keywords:** inflammatory labyrinthitis, infliximab, hearing disorders, vestibular diseases

## Abstract

Inflammatory labyrinthitis is defined as a fluctuant vestibulo-cochlear syndrome associated with an impairment of the blood-labyrinthine barrier (BLB) on delayed FLAIR MRI sequences. Systemic and intratympanic corticosteroids are the gold standard treatment but their effect is frequently insufficient. The objective is here to determine whether infliximab could be of value in the treatment of bilateral inflammatory labyrinthitis. A retrospective monocentric study was conducted between January 2013 and December 2021. All patients included in the study were affected with a bilateral vestibulo-cochlear syndrome associated with bilateral blood-labyrinthine barrier impairment. Patients were administered infliximab at the dose of 5 mg/kg every 6 weeks for 6 months. Audiometry, MRI with delayed FLAIR sequences on the labyrinth, and corticosteroid doses still required were assessed both before and after treatment with infliximab was completed. Pure-tone average (PTA) was the primary outcome. The secondary outcomes were the speech recognition threshold (SRT), the Dizziness Handicap Inventory (DHI) score, and the corticosteroid (CS) dose. A total of nine patients including five men and four women were enrolled in the study. Thirteen ears were analyzed. After a 6-month period of treatment, the mean PTA (54 ± 24 db versus 66 ± 22 db; *p* = 0.027), SRT (54 ± 37 db versus 66 ± 32 db; *p* = 0.041) and DHI score (27 ± 15 versus 9 ± 2; *p* = 0.032) significantly improved. After the 6-month treatment period, the mean CS dose decreased from 38 ± 33 to 6 ± 5 mg/day (*p* = 0.003). We conclude that infliximab substantially improves the vestibulo-cochlear function in patients with bilateral inflammatory labyrinthitis and could be of value in corticosteroid-dependent cases.

## 1. Introduction

The origin of both acute and fluctuant vestibulo-cochlear syndromes is often elusive. Infection, inflammation, and microvascular processes appear to be the three mechanisms by which the inner ear is affected while inflammation is thought to be the main cause of chronic syndromes [1]. Even though fluctuant vestibulo-cochlear deficits have often been considered auto-immune diseases of the inner ear, specific auto-antibodies have never been identified [2]. In most cases, an inflammatory process of the inner ear is likely. The clinical presentation includes a unilateral or bilateral vestibulo-cochlear deficit associated with an enhancement of the labyrinth on FLAIR sequences after injection of gadolinium. Inflammatory labyrinthitis is sometimes associated with systemic inflammatory diseases but can also be primary [3]. The disease is rather poorly described in the literature. Inflammatory labyrinthitis presents as an acute vestibulo-cochlear syndrome including unilateral or bilateral mild to profound sudden hearing loss, tinnitus, and rotatory vertigo [3]. At the time of diagnosis, the clinical examination would eliminate infectious labyrinthitis and neurological disorders. In our experience, the vestibular evaluation most often reveals a vestibular deficit including spontaneous nystagmus and impairment of the vestibular function on the video head impulse test, caloric testing, and vestibular evoked myogenic potentials. In its bilateral form, inflammatory labyrinthitis often presents with non-simultaneous bilateral vestibulo-cochlear symptoms. Impairment of the contralateral ear often occurs in the first year following the ipsilateral lesion. The evolution of inflammatory labyrinthitis is unpredictable: about 20% of the patients will develop chronic and fluctuant vestibulo-cochlear symptoms that need long-term treatment. Although corticosteroids (CS) by both systemic and trans-tympanic routes are considered to be the gold standard in the treatment of most vestibulo-cochlear syndromes, their effect is frequently insufficient [4]. Recurrent vertigo, chronic dizziness, and fluctuating or progressive hearing loss can persist for months in treated patients. In addition, CS dependence is frequent and can lead to severe complications over time.

Among inflammatory disorders of the inner ear, an auto-immune process against the hair cells of the cochlea has been evoked but a specific auto-antibody has been rarely identified [2,5,6]. An inflammatory cause is suspected when 3D-FLAIR MRI sequences and more specifically delayed FLAIR sequences after injection of contrast medium reveal an enhancement of the perilymph also known as blood-labyrinthine barrier (BLB) impairment [7]. However, enhancement of the perilymphatic space on delayed FLAIR sequences is not specific to inflammation as it could also be found in microvascular damage of the vestibulo-cochlear microcirculation. The identification of primary inflammatory labyrinthitis is therefore difficult and is based on an array of clinical data, imaging, and vestibulo-cochlear function testing.

The central role of tumor necrosis factor alpha (TNFα) in inflammation has been supported by the ability of agents that block the action of TNFα to treat a range of inflammatory conditions [8,9]. For instance, the monoclonal anti-TNFα antibody infliximab has been used for over 20 years to treat rheumatologic and gastrointestinal inflammatory disorders [8,9]. In the inner ear, the TNFα binding inhibitor etanercept has been evaluated for the treatment of auto-immune conditions [10,11,12]. In two of these studies, no significant improvement of the pure tone threshold (PTA) was observed after 8 weeks of treatment with etanercept. In these studies, the diagnosis of auto-immune inner ear disease relied on clinical and audiological criteria without the use of 3D-FLAIR MRI. Other studies have shown that anti-TNFα molecules were efficient in the treatment of sudden hearing loss associated with systemic inflammatory disorders [13,14,15] and in a recent meta-analysis, anti-rheumatologic drugs appeared to be effective in the treatment of chronic vestibulo-cochlear disorders [16]. Significant improvements in the pure-tone average (PTA) and the speech recognition threshold (SRT) were observed. Experimentally, etanercept has been proven to reduce inflammation of the inner ear after a local immunological aggression [17].

Our purpose here was to determine whether infliximab could be of value in the treatment of patients with inflammatory labyrinthitis.

## 2. Patients and Methods

*Patients and study characteristics*: a retrospective monocentric study was conducted between January 2013 and December 2021 in a tertiary care center in Paris, France. Patients with a bilateral vestibulo-cochlear syndrome associated with bilateral BLB impairment were included. Patients with a vestibulo-cochlear syndrome associated with unilateral BLB impairment and a history of severe or profound contralateral hearing loss were also included. Exclusion criteria were treatment contraindications, non-fluctuating hearing loss >3 months, isolated vestibular symptoms without hearing impairment, and age < 18. The absence of CS sensitivity was not an exclusion criterion. This study was carried out in accordance with the Declaration of Helsinki. Informed consent was obtained for each patient before treatment.

*Data*: data collected before treatment included age, gender, dose of CS before the onset of treatment, CS sensitivity and/or CS dependence of the vestibulo-cochlear symptoms, the total number of infusions received during follow-up, and delay of treatment. A screening for systemic diseases was performed for each patient and previous or ongoing anti-inflammatory treatments were reported. The level of CRP was used to assess the presence or absence of an inflammatory syndrome. MRI results, audiometric measurements and DHI scores were collected both before and after 6 months of treatment.

*Vestibulo-cochlear evaluation*: a complete neuro-otological examination was carried out before inclusion and every 6 weeks during follow-up. Audiograms were obtained every 6 weeks, before each infusion of infliximab. PTA was calculated as the average of the hearing thresholds at the frequencies of 500, 1000, 2000 and 4000 Hz. Hearing loss was classified according to PTA as mild (21–40 dB), moderate (41–70 dB), severe (71–90 dB) or profound (>90 dB). In the case of no-response, a threshold at 120 dB was established to allow statistical analysis. The Dizziness Handicap Inventory (DHI) was used to assess the patients’ quality of life before and after treatment. This 25-item scale provided a quality-of-life score ranging from 0–100 [18].

*Imaging*: MRI was carried out shortly before and 6 months after the onset of treatment; a 3T scanner (3T Siemens Skyra; Siemens Healthineers, Erlangen, Germany) with a 64-channel head–neck–spine coil was used. All patients received gadobrutol (Gd-DO3A-butrol, Gadovist 0.1 mmol/kg, 1 mmol/mL). A single intravenous dose of the contrast agent was administered 4 h before images were acquired. Blood-labyrinth barrier impairment was defined as an enhanced signal of the labyrinth on delayed FLAIR sequences. The diagnosis of BLB impairment was made by a neuroradiologist.

*Treatment*: infliximab was administered by infusion at the dose of 5 mg/kg for 6 months. The dose-loading phase consisted of three doses of infliximab (weeks 0, 2 and 6). The maintenance phase consisted of one dose on Infliximab every 6 weeks. Contraindications (chronic infections, cardiac insufficiency, history of malignant disease except for basal cell carcinoma, multiple sclerosis) were excluded before treatment. Subjects enrolled were allowed to continue CS; a progressive decrease was attempted according to the audiometric follow-up.

*Outcomes*: PTA was the primary outcome: an improvement in PTA was defined as a 10 dB gain compared to the pretreatment value. Secondary outcomes were the speech recognition threshold (SRT), the DHI scores, the degree of BLB impairment on MRI FLAIR sequences, and the dose of CS after 6 months of treatment. The side effects of treatment were also recorded.

*Statistical analysis*: all analyses were conducted with Prism 8.4.0 (Macintosh Version by Software MacKiev © 1994–2020 GraphPad Software, LLC, San Diego, CA, USA). Between-groups were compared using the Student t-test for parametric data and the Wilcoxon test for non-parametric data. Continuous values were expressed with their mean and standard deviation. Values were considered statistically significant when *p* < 0.05.

## 3. Results

*Patients’ characteristics*: they are reported in Table 1. A total of nine patients including five men and four women were enrolled in the study. Four of the patients presented with a bilateral vestibulo-cochlear syndrome associated with BLB impairment while the other five were affected with a unilateral vestibulo-cochlear syndrome associated with BLB impairment and a history of contralateral non-fluctuating severe or profound hearing loss. Only 13 of the 18 ears were analyzed; the remaining five ears had to be excluded because they had been functionally deaf for more than three months. All patients had hearing worsening or fluctuant hearing in one or both ears despite local and systemic CS at the time of enrollment.

BLB impairment was idiopathic in six patients. Two patients were diagnosed with Cogan syndrome and one patient with multi-visceral sarcoidosis. Baseline laboratory assessment including CRP was normal in eight patients. CRP was mildly elevated in one patient (CRP = 11 mg/L). The delay between the first symptom and the onset of treatment was 21 ± 22 months. Eight patients received all six infusions of infliximab. One patient received only five infusions because of iatrogenic hepatic cytolysis.

*Vestibulo-cochlear evaluation*: the results of the vestibulo-cochlear assessment before and after treatment and their statistical significance are reported in Table 2 and Table 3. Before treatment, all patients complained of tinnitus. Hearing loss was mild in three ears out of the 13 analyzed ears (23%), moderate in three ears (23%), severe in six ears (46%) and profound in one ear (8%). The mean PTA before treatment was 66 ± 22 db. There was no air–bone gap in any of the patients examined. The mean SRT before treatment was 66 ± 32 db.

**Figure 1:**
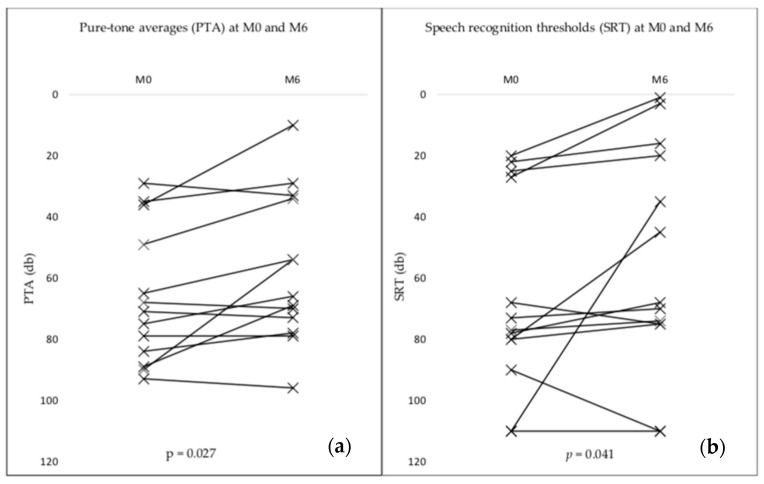
Pure-tone averages (PTA) and speech recognition thresholds (SRT) before treatment and at 6 months and statistical significance. (**a**) PTA (**b**) SRT.

After a 6-month period of treatment, tinnitus complaints disappeared in three out of the nine patients (33%) and persisted in six patients (67%). Hearing loss was mild in three ears (23%), moderate in five ears (38%), severe in three ears (23%) and profound in one ear (8%). On-ear recovered normal hearing at 6 months. The degree of hearing loss for each patient before and after treatment is reported in Table 4. The number of patients with a severe or profound hearing loss decreased from 54% to 31% after the 6-month treatment period. According to the criteria for hearing improvement as defined in the methods section, hearing was improved in five ears (38%) and stable in eight ears (62%). No patient experienced worsening of the PTA after treatment. The mean PTA (54 ± 24 db versus 66 ± 22 db; *p* = 0.027), SRT (54 ± 37 db versus 66 ± 32 db; *p* = 0.041) and the DHI score (27 ± 15 versus 9 ± 2; *p* = 0.032) significantly improved (Figure 1).

*Corticosteroids dose assessment*: seven patients were receiving long-term CS therapy at the onset of treatment and all patients had received trans-tympanic injections (TTI) of dexamethasone before the onset of treatment (mean 4 ± 1 TTI). The mean CS dose before treatment was 38 ± 33 mg/day. Symptoms were sensitive to CS in seven patients and resistant in two patients. Before treatment, six patients had CS-dependent symptoms. Two patients who had received an increased dose of CS before the onset of treatment received trans-tympanic injections of dexamethasone during treatment. After the 6-month treatment period, the mean CS dose significantly decreased to 6 ± 5 mg/day (*p* = 0.003). The decrease of the corticosteroid dose was allowed when hearing levels were stable measured by tonal and vocal audiometry.

**Figure 2:**
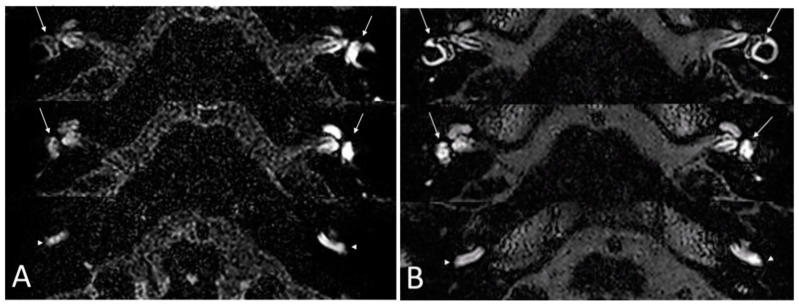
Delayed 3D-FLAIR MRI sequences. (**A**) Before treatment. Enhanced signal of the left vestibule (arrows) and the basal turn of the cochlea (arrowhead) compared with the right side. (**B**) At 6 months: symmetrical enhancement of the vestibule (arrows) and the basal turn of the cochlea (arrowhead).

*Imaging*: all patients received an MRI with FLAIR sequences on the labyrinth before treatment and at 6 months. Before treatment, BLB impairment affected the cochlea in all ears and the vestibule in 11 ears. After treatment, a decrease of the BLB impairment was noticed in all ears, defined as a partial or total regression of enhancement of the labyrinth on injected FLAIR sequences. There was no aggravation of BLB impairment during the clinical outcome in five ears. In six ears, an improvement of BLB impairment was seen without any improvement of the PTA. In two ears, there was no improvement in BLB impairment. Figure 2 illustrates an example of the improvement of the delayed FLAIR sequences on the labyrinth after the 6-month treatment period.

*Tolerance*: the treatment was interrupted at 6 months in one patient because mild hepatic cytolysis was observed. After 3 months of follow-up, hepatic cytolysis had regressed spontaneously. The treatment was not reintroduced after normalization of the transaminase levels. No other side effect was noted in our cohort during the 6 month-follow-up.

## 4. Discussion

This is the first study that investigates the effect of infliximab on the vestibulo-cochlear function of patients affected by inflammatory bilateral labyrinthitis with BLB impairment. Other studies have dealt with TNFα inhibitors and inflammatory inner ear disease but in these studies, molecules other than infliximab were used and results were often contradictory [10,11,12,19]. One explanation could lay in the different mechanisms of action of TNFα inhibitors: etanercept is a competitive inhibitor of TNFα binding to its receptor whereas Infliximab is an antibody directed against TNFα. These two molecules have proven to be of uneven efficacy in the treatment of gastrointestinal and rheumatologic inflammatory disorders [20]. In Cogan syndrome, infliximab is efficacious on vestibulo-cochlear symptoms, in contrast to disease-modifying anti-rheumatic drugs or steroids, which are not [21]. Part of the problem with previous investigations probably resulted from the poor selection of the cohort studied since the selection was based solely on clinical and audiological criteria. Here we were able to better select a potentially responsive population by using MRI of the internal auditory canals and specifically FLAIR sequences. Such imaging allowed us to discriminate patients with authentic inflammatory disorders among those with bilateral fluctuating vestibulo-cochlear syndromes. Identifying inflammatory labyrinthitis among fluctuant vestibulo-cochlear syndrome is challenging. We wish to emphasize here the importance of FLAIR sequences in the selection of patients who can benefit from anti-TNFα therapy. We noted that hearing levels improved in two out of the three patients who presented with inflammatory labyrinthitis secondary to a systemic disease (Cogan syndrome and sarcoidosis). In this case, an inflammatory process of the inner ear was very likely. Distinguishing inflammatory labyrinthitis from other causes of bilateral labyrinthitis could be the key to a reliable selection of patients that should be treated with infliximab. The identification of other mechanisms of inner ear disorders has not been achieved yet. Delayed FLAIR sequences on the labyrinth are more sensitive than usual FLAIR sequences and lead to more precise localization of inflammation. However, immediate FLAIR sequences can also be used as they can detect enhancement of the labyrinth after injection of contrast medium although with lesser accuracy of the topography of the inflammatory process. The development of delayed FLAIR sequences in the near future should allow for more precise diagnoses of disorders of the inner ear and should be the privileged imaging modality.

Our results were conclusive in terms of improvement in the pure-tone average, intelligibility threshold, subjective vestibular function, and quality of life. In our series, among 13 ears examined, five showed an improvement of the PTA of more than 10 db, and the improvement of BLB impairment was correlated with the improvement of the clinical vestibulo-cochlear outcome. As to the vestibular function, a significant improvement in the quality of life measured with the DHI was noted after 6 months of treatment with infliximab. We noticed that hearing levels were improved in only five out of 13 ears, but significant improvement was achieved for those. This poses the question of a more precise selection of the patients that would benefit from infliximab. However, no predictive factor of good response could be identified in this study. A larger cohort could be of value to assess predictive factors. Treatment with infliximab led to CS sparing in all patients. The mean CS dose at 6 months could be diminished in all the patients treated with infliximab and all patients had a CS dose inferior to 20 mg/day after 6 months of treatment. The absence of CS dependence before treatment did not seem to be o predictive factor of non-response. The decrease in the CS dose is a major improvement in the management of this disease since hearing fluctuations are frequent and need iterative dose adaptations that lead to long-term treatments. In patients with bilateral disease, hearing preservation is a priority despite the toxicity of CS.

Recent hearing fluctuations and vertigo or dizziness despite systemic steroid treatment are the two conditions to consider when introducing infliximab in the course of the disease. The optimal timing of treatment with infliximab could not be determined in our study, but the monoclonal antibody should be considered rapidly in case of CS dependence or CS treatment failure. We could confirm that infliximab was a safe modality treatment since few side effects were observed. Treatment had to be discontinued in only one patient for transient hepatic cytolysis. These data were in agreement with the literature [22]. No severe or long-term side effects were observed during the follow-up.

The first limitation of the study presented here is the small number of patients included: only 13 ears were analyzed. Bilateral disorders of the inner ear are rare, and anti-TNFα therapy needs a precise selection of the patients to be treated. A larger cohort and a randomized controlled trial are required to confirm the results presented here. Also, long-term outcomes need to be analyzed since this is a chronic fluctuant disorder. One cannot exclude that random fluctuations at the time of the outcome evaluation might have distorted our results to some extent. However, an early evaluation at 6 months seemed to be relevant. In gastrointestinal and rheumatologic inflammatory disorders, the efficacy of infliximab has often been evaluated at 6 months and was found to be predictive of the long-term response [23]. A vestibulo-cochlear examination at 3 to 5 years would consolidate the present data. The evaluation of fluctuant disorders is often subject to important biases due to the unpredictable evolution of symptoms and the outcome can be dependent on the time at evaluation. However, the improvement of the blood-labyrinthine impairment on delayed FLAIR sequences was an argument in favor of a long-term improvement of the disease.

We find that infliximab is particularly promising as an efficient and safe option for the treatment of chronic vestibulo-cochlear syndromes associated with BLB impairment. Infliximab should be considered in patients who fail to respond to CS or show CS dependence. Because of the chronic and fluctuating nature of the disease, consensus needs to be obtained as to the duration of treatment in case of stabilization of the vestibulo-cochlear function. Using infliximab for the treatment of unilateral inflammatory disorders of the inner ear might be the next step to treat severe or profound vestibulocochlear deficits that are responsible for a severe alteration of the quality of life.

## Figures and Tables

**Table 1:** Patients’ characteristics before treatment.

Patient	Age (y)	Gender	CRP (mg/L)	Systemic Disease	CS Dose (mg/d)	CS Sensitivity	CS Dependence	PTA (dB)	SRT (dB)	DHI
1	25	Female	4	no	15	no	yes	29	22	58
2	35	Male	4	Cogan syndrome	70	yes	no	84	80	26
						yes	no	65	80	
3	28	Male	4	no	80	yes	no	93	90	26
4	22	Male	11	no	80	yes	yes	49	27	16
5	25	Female	4	Cogan syndrome	60	yes	yes	90	110	20
						yes	yes	36	20	
6	55	Female	0	no	0	yes	yes	79	77	10
						yes	yes	68	78	
7	54	Male	4	no	0	no	no	75	68	36
						no	no	35	25	
8	30	Male	1	no	9	yes	yes	71	73	38
9	41	Female	4	Sarcoidosis	60	yes	yes	89	110	12

CS: corticosteroid. PTA: pure-tone average. SRT: speech recognition threshold. CRP: C-reactive protein. DHI: dizziness handicap inventory.

**Table 2:** Vestibulo-cochlear outcomes after treatment.

Outcome	Tinnitus (*n* = 9)	Hearing Loss (*n* = 13)	Vertigo (*n* = 9)	BLB Impairment (*n*= 13)
Improved (%)	3 (33)	5 (38)	8 (87)	13 (100)
Stable (%)	6 (66)	8 (62)	1 (13)	0 (0)
Worse (%)	0 (0)	0 (0)	0 (0)	0 (0)

**Table 3:** Comparative analysis of PTA, SRT, DHI, and CS dose before and after treatment.

Outcome	Before Treatment	After 6 Month-Treatment	*p*
PTA (dB)	64 ± 22	53 ± 23	**0.027**
SRT (dB)	66 ± 32	49 ± 33	**0.041**
DHI	27 ± 15	9 ± 2	**0.032**
CS dose (mg/day)	38 ± 33	6 ± 5	**0.003**

Statistically significant values are in bold.

**Table 4:** Degree of hearing loss before and after treatment for each patient.

Patient	Hearing Loss	
	Before Treatment	After Treatment
1	mild	mild
2	severe	severe
	moderate	moderate
3	profound	profound
4	moderate	mild
5	severe	moderate
	mild	normal hearing
6	severe	severe
	moderate	moderate
7	severe	moderate
	mild	mild
8	severe	severe
9	severe	moderate

## Data Availability

The data presented in this study are available on request from the corresponding author.
